# Understanding diabetes risk in the Y Community of Greater Brisbane: Findings from a cross‐sectional survey

**DOI:** 10.1002/hpja.889

**Published:** 2024-06-12

**Authors:** Lucy E. Campbell, Sjaan R. Gomersall, Michael Tsiamis, Ana D. Goode, Genevieve N. Healy

**Affiliations:** ^1^ The University of Queensland, School of Human Movement and Nutrition Sciences, Health and Wellbeing Centre for Research Innovation Brisbane Australia; ^2^ The University of Queensland, School of Health and Rehabilitation Sciences Brisbane Australia; ^3^ The Y Queensland Fortitude Valley Australia

**Keywords:** community needs assessment, cross‐sectional survey, diabetes, prevention program, preventive health, type 2 diabetes mellitus

## Abstract

**Background:**

This cross‐sectional study aimed to understand the need and desire for a diabetes prevention program within the Y (formerly YMCA: Young Men's Christian Association) of the Greater Brisbane region, Queensland, Australia.

**Methods:**

An anonymous online survey was distributed (March–April 2023) by The Y Queensland targeting adults within the Greater Brisbane Y community. Data were collected on Y membership and branch attended, postcode, diabetes risk in the next 5 years (low, medium, or high), and interest in participation in a diabetes prevention program. Data were analysed via descriptives and cross tabulation with statistical significance considered at *p* < .05.

**Results:**

Respondents (*n* = 575) were primarily female (65%), attending a Y branch located in the outer city (51%), and aged under 55 years (68%). Twenty Y sites were represented, with a mix of inner‐city, outer‐city, and regional areas. Overall, 46% (*n* = 241/530) of respondents were at high diabetes risk, with those living in relatively socio‐economically disadvantaged areas more likely (*p* < .001) to be at high‐risk (57%) than intermediate (26%) or low‐risk (18%). Most (68%) respondents were interested/potentially interested in program participation; those at high risk of developing diabetes in the next 5 years were most interested (55%).

**Conclusions:**

The Y in Greater Brisbane may provide a suitable setting to host a community‐based diabetes prevention program. Locations outside the inner city should be prioritised to target those who are relatively socio‐economically disadvantaged to align with higher need and demand.

**So What?:**

Findings inform the implementation and prioritisation of a community‐delivered diabetes prevention program.

## 1 INTRODUCTION

Chronic health conditions such as cardiovascular disease, hypertension and type 2 diabetes mellitus are experienced by almost half of the Australian population (47% in 2017–2018),^1^ with corresponding health, financial and social impacts for both individuals and broader society.^2^ The seminal Diabetes Prevention Programs demonstrated that lifestyle interventions can prevent diabetes development in individuals at high risk or with pre‐diabetes,^3^, ^4^ with these beneficial effects persisting a decade post intervention.^5^, ^6^ These interventions involved supporting participants to eat healthier, engage in physical activity of at least moderate‐intensity activity, and to achieve and maintain weight loss through personalised support and coaching from trained professionals.^4^ However, attempts to scale up from these original intensive interventions and create sustainable diabetes prevention programs within communities have met with challenges, including program reach and retention of participants^7^ as well as loss of fidelity and reductions in efficacy.^8^ Additionally, translation programs have struggled to serve populations in both low socio‐economic and highly ethnically diverse areas,^7^ which are often the communities which are at greatest risk of type 2 diabetes.^9^ As such, new thinking about the design and delivery of programs has been required to create diabetes prevention programs that reach and serve the populations in most need and that are sustainable and practical over the long term and in ‘real‐world’ conditions.

One response has been the delivery of diabetes prevention programs in community‐based settings that are already well accepted and established. Translations of both the US and Finnish diabetes prevention programs have been implemented in a variety of settings including churches^10^ and YMCAs (Young Men's Christian Association).^11^, ^12^, ^13^ YMCAs are an ideal setting for diabetes prevention programs that seek to address the needs of underserved populations as they have significant community reach and financial assistance programs to subsidise the cost of programs for low‐income groups.^14^ The YMCA has hosted diabetes prevention programs in both the United States, where the National Diabetes Prevention Program was adapted specifically to be placed into YMCAs across the country,^11^, ^14^ and in Canada which has more recently seen the Small Steps for Big Changes diabetes prevention program implemented in YMCA sites in British Columbia.^13^ Both programs demonstrated effectiveness,^11^, ^13^ suggesting that the YMCA is a suitable delivery site for diabetes prevention programs.

In line with the figures for Australia more generally, Greater Brisbane, in South‐East Queensland Australia, is experiencing unsustainable rates of type 2 diabetes.^15^ The Y Queensland (formerly the YMCA with the rebranding to The Y intended to reflect that the organisation supports everyone, regardless of age, gender, religion, sexuality or difference) has sites across Greater Brisbane, serving areas across a range of socio‐economic status and with wide cultural diversity. It is thus likely that The Y will be a suitable site for delivery of a diabetes prevention program. However, prior to investment in such a program, there needs to be understanding of both the need and the desire for such a program within the community. Conducting a local needs assessment has been identified as an important implementation planning strategy to help inform ‘how’ to deliver a diabetes prevention program in the local community.^16^, ^17^ Correspondingly, the aims of this study were to determine the level of risk for developing type 2 diabetes in adults associated with The Y in Greater Brisbane in the next 5 years, and to understand the willingness of potential participants from this community to engage with a healthy lifestyle type 2 diabetes prevention program.

## 2 METHODS

This cross‐sectional study was conducted in collaboration with The Y Queensland and The University of Queensland. This study received ethical approval from The University of Queensland Human Research Ethics committee (2022/HE002421) and all participants provided informed consent before completing the survey. Eligible participants were adults aged over 18 years, affiliated in any way with The Y and reached by their survey recruitment drive. Recruitment was conducted via convenience sampling in collaboration with The Y and comprised advertisements delivered via mailing lists and social media (including Facebook and Instagram). Participants were invited to opt into a random draw to win one of three $100 Coles/Myer Gift Cards.

Data collection comprised an online anonymous survey hosted through the Qualtrics platform with a one‐month data collection period (24th March 2023 to 24th April 2023). The survey questions included a mix of yes/no answers, check boxes, and free text short answer questions and was estimated to take less than 5 min to complete. Data were collected on current membership status of The Y, branch they attend/attended, and level of interest in participating in a diabetes prevention program (interested/potentially interested/not interested/prefer not to say). Risk of developing diabetes in the next 5 years was assessed using the AUSDRISK tool^18^ with permission from the Australian Government Department of Health and Aged Care. The AUSDRISK tool comprises a series of questions about diabetes risk factors. These include age, sex, personal and family history of high blood glucose, First Nations background (Aboriginal, Torres Strait Islander, Pacific Islander and Māori), other ethnic minority background (Asian, Middle Eastern, North African and Southern European background), if taking medication for hypertension, smoking status, diet, exercise habits, and waist measurement. A diabetes risk score between zero and 38 is then calculated with scores being assigned according to risk for each factor where a higher score indicates a higher risk of developing type 2 diabetes.^18^ The AUSDRISK tool was developed using data from the 1999–2000 Australian Diabetes, Obesity and Lifestyle Study (AusDiab)^19^ and is a simple but valid and reliable risk assessment tool to predict incident type 2 diabetes within the next 5 years.^20^ Participants were also given the opportunity to provide any other comments via a single open‐text field.

Recoding occurred for four quantitative variables: location of Y branch attended, waist measurement, high risk ethnic background and diabetes risk score. Y branch locations were recoded into either inner city, outer city, or regional. Inner city locations were determined on classifications from Brisbane City Council,^21^ outer city locations fell within either Brisbane City, Logan City, Ipswich City, Redland City or Moreton Bay Regional Council areas. The two regional Y branches were outside the boundary of the above Council areas (see Supplementary Table 1 for a list of all Y branches included in this study and their categorisation or inner city, outer city or regional). Waist measurement was recoded into a low‐risk, medium‐risk and high‐risk category according to the risk score associated with measurement groupings on the AUSDRISK tool. Waist measurement scoring on the AUSDRISK tool is dependent on both sex and ethnic minority status with different ranges provided depending on if the individual is male or female, or if they have First Nations, Asian, Middle Eastern, North African or Southern European heritage.^18^ High‐risk ethnic background was a recoded variable which combined First Nations background (Aboriginal, Torres Strait Islander, Pacific Islander and Māori) with Asian, Middle Eastern, North African and Southern European backgrounds. Individual diabetes risk scores were recoded to be grouped into low (a score of five or below), intermediate (a score of six to 11) and high risk (a score of 12 and over) categories. Rating of advantage and disadvantage was derived from postcode data using the index of relative socio‐economic advantage and disadvantage (IRSAD) provided by the Australian Bureau of Statistics.^22^ A rating of 1 indicates the highest level of relative socio‐economic disadvantage and a rating of 5 indicates the highest level of relative socio‐economic advantage.^22^ These five quintiles were then collapsed into two categories: relatively socio‐economically disadvantaged (quintiles one to three), and relatively socio‐economically advantaged (quintiles four and five). Interest in a diabetes prevention program was collapsed to interested or potentially interested/not interested or prefer not to say.

Demographic characteristics and quantitative variables were analysed using descriptive statistics via SPSS version 29.0 (IBM, New York USA). Further analysis was conducted using cross tabulation and Pearson chi‐square tests to understand the demographic profile (location, age, sex, level of advantage and disadvantage and ethnic minority status) of those interested and not interested in a diabetes prevention program hosted by The Y. The same tests were performed for interest in a diabetes prevention program, and location according to diabetes risk score category (low, intermediate or high). A diabetes risk score was not able to be calculated for respondents who answered ‘prefer not to say’ for ethnic minority background (*n* = 1), smoking (*n* = 2) and waist measurement (*n* = 15) in addition to those who answered ‘unsure’ for family history of diabetes (*n* = 6) and as such these participants were excluded where risk scores were required for analyses. Open text data were analysed using thematic analysis.^23^

## 3 RESULTS

The survey had a reach of approximately 13 500 people (13 000 via email lists, and another 500 via Facebook and Instagram), of which 642 responded and 638 consented. The estimated reach does not include those who were made aware of the study by friends or family, and similarly the number of responses does not include those who clicked on the survey and then closed the browser. A total of 63 respondents did not complete any questions related to diabetes risk, leaving 575 responses with the majority of the survey completed. From these, a valid AUSDRISK score was able to be calculated for 530 respondents based on the available data. A total of 633 respondents provided their Y membership status of whom 86% (*n* = 531) were current or past members, with the majority attending a Y branch either in the outer city (*n* = 270, 51%) or regional area (*n* = 55, 10%), rather than inner city Brisbane (*n* = 206, 39%). According to IRSAD data, three quarters of respondents (*n* = 419/559, 75%) live in an area considered relatively socio‐economically advantaged.

Table 1 outlines the results of the frequency analysis of the AUSDRISK variables. Approximately half of respondents were under the age of 45, and a greater proportion were female (65%), with no participants reporting intersex variations. Just under one third (157; 29%) reported an ethnic background of high diabetes risk, that is, an Aboriginal, Torres Strait Islander, Pacific Islander, Māori, Asian, Middle Eastern, North African or Southern European background.

**TABLE 1:** Diabetes risk factors asked by the AUSDRISK tool and approximate risk of developing diabetes within the next 5 years.

Measure	Frequency (*n*)	Percent (%)
Age (*n =* 575)		
Under 35 years	154	26.8
35 to 44 years	135	23.5
45 to 54 years	103	17.9
55 to 64 years	84	14.6
65 years and over	99	17.2
Sex (*n* = 575), Female	372	64.7
First Nations descent (*n* = 575), yes	103	17.9
Ethnic minority background (*n* = 574), yes	111	19.3
Diabetes diagnosis in immediate family (*n* = 569), yes	223	39.2
History of high blood glucose (*n* = 575), yes	131	22.8
Medicated for hypertension (*n* = 575), yes	127	22.1
Daily tobacco smoking (*n* = 573), yes	93	16.2
Daily consumption of fruits and vegetables (*n* = 575), yes	441	76.7
Reach 150 min of physical activity per week (*n* = 575), yes	430	74.8
Waist measurement (*n =* 570)		
High risk	84	14.7
Medium risk	191	33.5
Low risk	280	49.1
Prefer not to say	15	2.6
Diabetes risk score (*n* = 530)		
High Risk (score ≥ 12)	241	45.5
Intermediate Risk (score 6 to 11)	187	35.3
Low Risk (score ≤5)	102	19.2
High risk category scores (*n* = 241) and risk for diabetes		
12 to 15 (~1 in 14 in next 5 years)	94	39.0
16 to 19 (~1 in 7 in next 5 years)	53	22.0
≥20 (~1 in 3 in next 5 years)	94	39.0

*Note*: First nations descent includes Aboriginal, Torres Strait Islander, Māori and/or Pacific Islander. Ethnic minority background was a place of birth or ethnic background from Asia, the Middle East, North Africa or Southern Europe. Immediate family includes parents and siblings.

In terms of genetic and medical risk factors 263 (41%) respondents indicated that an immediate family member had been diagnosed with diabetes and/or they themselves had a history of high blood glucose, and 127 (22%) were currently medicated for hypertension. In relation to lifestyle risk factors of daily tobacco smoking, not consuming fruits and vegetables daily, and not meeting the recommended 150 min of weekly physical activity, 401 (63%) reported having none of these risk factors, 149 (23%) reported one, 53 (8%) reported two and 39 (6%) reported all three risk factors. When focusing on the two lifestyle risk factors most commonly targeted in diabetes prevention programs (physical activity and diet) most reported having neither risk factor (68%), while 21% reported one risk factor, and 11% reported two. Almost half (48%) of respondents had a waist measurement which fell into either the medium‐risk or high‐risk category for their respective sex and ethnic background. Overall, a valid diabetes risk score was able to be calculated for 530 respondents with a median score of 11 and interquartile range of 10. Of these, the majority (55%) were at a low or intermediate risk of developing type 2 diabetes within the next 5 years. From the 241 respondents whose diabetes risk score fell into the high‐risk category (12 and over), most respondents (147; 61%) received a diabetes risk score of 16 or more.

The majority of respondents (68%) were either interested or potentially interested in a healthy lifestyle diabetes prevention program. Table 2 shows the interest by demographic characteristics. Interest in program participation was significantly associated with age (*p* = .028), where those aged over 65 years were more likely to be interested or potentially interested (79%) compared to the total across all age groups (68%). Interest also varied by ethnic background, with 87% of respondents with high‐risk ethnic ancestry interested or potentially interested in a diabetes prevention program which was significantly (*p* < .001) higher than the total of all respondents (68%). Interest did not vary significantly or meaningfully (all variations less than 10%) for Y Branch location, sex, or level of disadvantage.

**TABLE 2:** Demographic data of respondents according to level of interest in a diabetes prevention program.

	Overall	Interested	Not interested	*p*
Y Branch Location	480	323 (67.3)	157 (32.7)	.213
Inner city	187 (39.0)	120 (64.2)	67 (35.8)	
Outer city	244 (50.8)	173 (70.9)	71 (29.1)	
Regional	49 (10.2)	30 (61.2)	19 (38.8)	
Age	548	373 (68.1)	175 (31.9)	.028
Under 35	146 (26.6)	92 (63.0)	54 (37.0)	
35 to 44	132 (24.1)	83 (62.9)	49 (37.1)	
45 to 54	97 (17.7)	64 (66.0)	33 (34.0)	
55 to 64	81 (14.8)	61 (75.3)	20 (24.7)	
65 and over	92 (16.8)	73 (79.3)	19 (20.7)	
Sex	548	373 (68.1)	175 (31.9)	.199
Female	358 (65.3)	237 (66.2)	121 (33.8)	
Male	190 (34.7)	136 (71.6)	54 (28.4)	
IRSAD	513	345 (67.3)	168 (32.7)	.101
Relative disadvantaged	130 (25.3)	95 (73.1)	35 (26.9)	
Relative advantaged	383 (74.7)	250 (65.3)	133 (34.7)	
High‐risk ethnic minority	547	373 (68.2)	174 (31.8)	<.001
Yes	157 (28.7)	137 (87.3)	20 (12.7)	
No	390 (71.3)	236 (60.5)	154 (39.5)	

*Note*: Values are reported as *N* (%). High‐risk ethnic minority status includes Aboriginal, Torres Strait Islander, Pacific Islander, Māori, Asian, Middle Eastern, North African and/or Southern European ancestry. *p*‐Values calculated using the Pearson chi‐square test. Interested included interested and potentially interested; not interested includes not interested and prefer not to say.

Abbreviation: IRSAD, index of relative socio‐economic advantage and disadvantage.

Table 3 shows the relationship of diabetes risk score with interest in a diabetes prevention program. Respondents who scored as high‐risk were significantly (*p* < .001) more likely to be interested in participating in a diabetes prevention program (55%) compared to those who received a low‐risk score (11%). Y branch location also had a significant relationship with diabetes risk score (*p* < .01), where respondents who were high risk were more likely to attend an outer city Y branch (51%) and less likely to attend an inner‐city Y branch (35%) compared to the overall percentage with a high risk (44%). Similarly, the place in which respondents lived was significantly associated with diabetes risk score (*p* < .001). Respondents who reported living in a relatively disadvantaged area were more likely to score in the high‐risk category for diabetes risk score (57% compared to the total for the high‐risk category of 42%), and less likely to receive a score in the intermediate category.

**TABLE 3:** Proportion of respondents in high, intermediate and low diabetes risk score categories according to interest in a prevention program, location and IRSAD.

Measures	Overall	High risk (≥12)	Intermediate (6–11)	Low risk (≤5)	*p*
DPP interest	511	231 (45.2)	181 (35.4)	99 (19.4)	<.001
Interested	361 (70.6)	198 (54.8)	123 (34.1)	40 (11.1)	
Not interested	150 (29.4)	33 (22.0)	58 (38.7)	59 (39.3)	
Y Branch location	463	203 (43.8)	173 (37.4)	87 (18.8)	.005
Inner city	180 (38.9)	63 (35.0)	72 (40.0)	45 (25.0)	
Outer city	237 (51.2)	121 (51.1)	85 (35.9)	31 (13.1)	
Regional	46 (9.9)	19 (41.3)	16 (34.8)	11 (23.9)	
IRSAD	495	210 (42.4)	186 (37.6)	99 (20.0)	<.001
Relatively disadvantaged	125 (25.3)	71 (56.8)	32 (25.6)	22 (17.6)	
Relatively advantaged	370 (74.7)	139 (37.6)	154 (41.6)	77 (20.8)	

*Note*: All values are reported as *N* (%). *p*‐Values calculated using the Pearson chi‐square test. Interested included interested and potentially interested; not interested includes not interested and prefer not to say.

Abbreviations: DPP, diabetes prevention program; IRSAD, index of relative socio‐economic advantage and disadvantage.

Seventy five people responded to the open text question. Overall, the primary theme that emerged was that this community is supportive of a diabetes prevention program. Responses then tended to fall into two sub‐themes: seeking more information about diabetes and diabetes prevention; and interest in the practicalities of a prevention program. In terms of the first sub‐theme, many respondents expressed curiosity about how individual risk factors determined overall diabetes risk, if certain other health conditions were also risk factors (such as polycystic ovary syndrome and attention deficit hyperactivity disorder), and where to find more information about diabetes prevention. The sub‐theme regarding practicalities of a prevention program included queries about what a prevention program would entail, and suggestions for what they would like to see included (such as wanting diet suggestions, how to manage weight, and how to lose weight from around the waist). For example, one respondent wrote ‘Recently diagnosed with pre‐diabetes. So would be very interested. Y could offer simple blood sugar tests to help clients identify if they need to seek medical advice’. Another asked, ‘Can the program be pursued at home? Or is gym equipment required?’ Both quotes suggest that respondents are already considering what the process of participating in a diabetes prevention program will look like for them.

## 4 DISCUSSION

In recognition of the unsustainable burden of type 2 diabetes in the Greater Brisbane area, this study examined the need and desire for a diabetes prevention program to be delivered through The Y branches in this community. Having a local needs assessment is important to make informed decisions about a future community diabetes prevention program. Findings showed that there was both a need, with nearly half (46%) of respondents at high risk of developing diabetes in the next 5 years, and a desire, with 71% of respondents at least potentially interested in a diabetes prevention program and the open text responses providing further indication of interest. Further to this, an encouraging outcome was that those who fell into the high‐risk category were also significantly more likely to be interested in a prevention program. These results suggests that respondents were willing to engage in lifestyle changes in order to improve their overall health and reduce their diabetes risk. The relationship between knowledge of diabetes risk and therefore readiness to engage with lifestyle changes has already been demonstrated,^24^ but this outcome was encouraging for consideration in the local context.

Findings demonstrated a significant association between diabetes risk and both Y branch location and the relative socio‐economic advantage and disadvantage of the area respondents lived in. Previous research has shown that household socio‐economic status is a strong predictor of incident type 2 diabetes in an Australian context, and that risk can be mediated by the presence of certain health behaviours such as not smoking and engaging in physical activity.^25^ Similarly, and more relevant for the data collected during this study, the relative disadvantage of the area in which an individual lives has a significant impact on the development of abnormal glucose metabolism (impaired fasting glucose, impaired glucose tolerance and type 2 diabetes) regardless of individual socio‐economic status.^26^ This association can be explained by the impact of environmental factors on diabetes incidence such as accessibility, attractiveness, and safety of outdoor space for exercise, access to high‐quality healthy food options and density of fast food outlets within a neighbourhood.^26^ While the majority of respondents within this study were considered relatively advantaged (IRSAD quintiles four and five derived from their postcode data), the differences between inner and outer city Y locations in terms of diabetes risk may be more informative about the kinds of healthy lifestyle resources available to The Y community in those areas.

The demographic profile of those interested in a diabetes prevention program is an important consideration given that underserved, socio‐economically disadvantaged communities are often at most need of diabetes prevention initiatives as noted previously.^26^ There was no significant or large impact on diabetes prevention program interest according to Y location and relative socio‐economic advantage/disadvantage, although among those who lived in relatively disadvantaged areas (outer city locations, and IRSAD one, two and three), more respondents expressed interest in such a program than disinterest. This suggests that people in these areas are looking for the programs and resources to help make changes in their lifestyle behaviours. The two demographic variables which were significantly associated with program interest were age and high‐risk ethnic background. Age was only significant for those aged 65 and over who were more likely to be interested in engaging in a diabetes prevention program than the average across all age groups. The surprising but encouraging finding from this study was the relationship between coming from a high‐risk ethnic background and interest in a diabetes prevention program (*p* < .001). Respondents whose ethnic background places them at higher risk of diabetes were much more likely to express interest in a prevention program (87%) than state that they were not interested (13%). If the results from this study are directly translated to diabetes prevention program recruitment by The Y in Greater Brisbane, the proportion of ethnic minority participants would potentially be encouragingly high, particularly if the recruitment and intervention materials were co‐developed with these communities to be culturally appropriate, and delivery agents were trained to be culturally safe.^27^

Implications for a possible diabetes prevention program in Greater Brisbane arising from this study are two‐fold. First, The Y is likely a suitable host for a diabetes prevention program in Brisbane due to several factors: it has the relevant resources already in place in terms of space, equipment and human resources; The Y community seems to be interested in health and wellbeing generally and education and programs on diabetes prevention more specifically and, The Y has demonstrated that it has strong connections to ethnic minority populations which will hopefully translate to program recruitment within this population. Second, opportunities outside the inner city should be prioritised to target those who are relatively socio‐economically disadvantaged and therefore are potentially limited in terms of healthy lifestyle resources.

There are two major limitations to this study. First, the self‐report nature of the survey may encourage social desirability bias^28^ and have a lower validity when compared to direct measures. Second, respondents to this survey are likely to be the portion of The Y community that are already interested in type 2 diabetes and its prevention which again can introduce bias. Due to both limitations the results presented here should be treated with caution. Somewhat mediating the above limitations however are the strengths of this study being the relatively strong response from the Y community with 642 overall responses, and the use of a valid and reliable diabetes risk assessment tool (AUSDRISK) which is widely used within the Australian context,^20^ despite its self‐report status.

An area of further research is the role of AUSDRISK as an eligibility measure for a diabetes prevention program hosted by The Y. While AUSDRISK is a widely utilised tool, and the ease of its use was demonstrated in this study, its efficacy has been questioned as a screening tool for entry into lifestyle modification interventions.^29^ One recommendation that has been suggested is that an AUSDRISK score of 15 or above be used in conjunction with a fasting plasma glucose or HbA1c test for recruitment into a diabetes prevention program.^30^ Further research could determine what the proportion of the Y community in greater Brisbane would meet this eligibility threshold, or if for ease of recruitment (i.e., not requiring a blood test) The Y should limit eligibility requirements to an AUSDRISK score alone.

## 5 CONCLUSION

This study determined that there is both a need and a desire for a diabetes prevention program among the community serviced by The Y Greater Brisbane. If such a program is offered, The Y should ensure that delivery sites include those outside of major metropolitan areas to target relatively disadvantaged populations who are at greater risk of developing diabetes. Further research should include how The Y will manage eligibility into a diabetes prevention program.

## AUTHOR CONTRIBUTIONS

**LC**: Conceptualisation; methodology; formal analysis; investigation; data curation; writing—original draft preparation; writing—review & editing; visualisation. **SRJ**: Conceptualisation; methodology; writing—review & editing; supervision. **ADG**: Writing—review & editing; supervision; project administration. **MT**: Resources; writing—review & editing; project administration. **GNH**: Conceptualisation; methodology; writing—review & editing; supervision.

## FUNDING INFORMATION

LC was supported by the University of Queensland Summer Scholarship. GNH is supported by an NHMRC/MRFF Emerging Leadership Fellowship. SRG is partly funded by the Health and Wellbeing Centre for Research Innovation, which is co‐funded by The University of Queensland and Health and Wellbeing Queensland.

## CONFLICT OF INTEREST STATEMENT

The authors declare no conflicts of interest.

## ETHICS STATEMENT

The project received ethical approval from The University of Queensland Human Research Ethics committee (2022/HE002421) and all participants provided informed consent.

## Supporting information


**SUPPLEMENTARY TABLE 1.** Descriptive statistics of overall DPP interest, all Y branches and IRSAD.

## Data Availability

The data that support the findings of this study are available on request from the corresponding author. The data are not publicly available due to privacy or ethical restrictions.
