# Supplementary material for: Understanding diabetes risk in the Y Community of Greater Brisbane: Findings from a cross‐sectional survey
Source: Health Promot J Austr. 2024 Jun 12;36(1):e889. doi: 10.1002/hpja.889 (PMC11729537; doi:10.1002/hpja.889)
Supplement: Supplementary file 1 — SUPPLEMENTARY TABLE 1. Descriptive statistics of overall DPP interest, all Y branches and IRSAD. [file HPJA-36-0-s001.docx]

| **Supplemental Table 1:** Descriptive statistics of overall DPP interest, all Y branches and IRSAD | | |
| --- | --- | --- |
|  | **Frequency** | **Percentage** |
| **DPP Interest (n=548)** |  |  |
| Interested | 373 | 68.1 |
| Not interested | 175 | 31.9 |
| **Y branch location (n=535)** |  |  |
| **Inner city** |  |  |
| Bowen Hills | 109 | 20.4 |
| Brisbane CBD | 34 | 6.4 |
| Kelvin Grove | 59 | 11 |
| Gardens Point | 4 | 0.7 |
| **Outer city** |  |  |
| Acacia Ridge | 11 | 2.1 |
| Cannon Hill | 7 | 1.3 |
| Ipswich | 10 | 1.9 |
| Jamboree Heights | 81 | 15.1 |
| Jindalee | 1 | 0.2 |
| Mango Hill | 28 | 5.2 |
| Northlakes | 7 | 1.3 |
| Redlands | 13 | 2.4 |
| Springfield Central | 7 | 1.3 |
| Springfield Lakes | 5 | 0.9 |
| Stafford | 11 | 2.1 |
| Victoria Point | 86 | 16.1 |
| Wynnum | 1 | 0.2 |
| Yarrabilba | 2 | 0.4 |
| Unsure | 4 | 0.7 |
| **Regional** |  |  |
| Caloundra | 19 | 3.6 |
| Warwick | 36 | 6.7 |
| **IRSAD (n=559)** |  |  |
| 1 (most disadvantaged) | 55 | 9.8 |
| 2 | 31 | 5.5 |
| 3 | 54 | 9.7 |
| 4 | 117 | 20.9 |
| 5 (most advantaged) | 302 | 54 |
| IRSAD = Index of Relative Socio-economic Advantage and Disadvantage. DPP = Diabetes Prevention Program | | |
